# THE EFFECT OF SENSE OF COHERENCE ENHANCEMENT PROGRAM BASED ON SALUTOGENESIS FOR ELDERLY WITH METABOLIC SYNDROME

**DOI:** 10.1093/geroni/igz038.513

**Published:** 2019-11-08

**Authors:** Jungmi Yun, Hyoung Sook Park

**Affiliations:** College of Nursing, Pusan National University, Busan, Korea, Republic of

## Abstract

This study aimed to develop and verify a sense of coherence (SOC) enhancement program based on salutogenesis for elderly with metabolic syndrome. This study was a quasi-experimental study. The inclusion criteria were patients with metabolic syndrome aged 60 or over who visited the outpatient department of a university hospital and a public health center located in an urban area. The intervention group (n=24) attended the SOC enhancement program. The health promotion theoretical framework, salutogenesis, provided the program structure and conceptual framework of this study. This program included comprehensibility, manageability, and meaningfulness which were core concepts of salutogenesis and consisted of multiple tailored behavior interventions composed of education, Tai-Chi exercises, individual activities, self-monitoring, self-help groups, and individual counseling. This program was provided once a week over 10 weeks, but Tai-Chi exercise was conducted twice a week. The control group (n=25) was provided with general health information once. The interactions between groups and measurement time showed significant differences in self-efficacy (p=.001), perceived social support (p<.001), SOC (p<.001), health behavior (p=.010), and the physical health-related quality of life (p=.001). The metabolic-related physiological indicators showed significant improvement in total cholesterol (p=.004) and LDL-cholesterol (p=.009). This study showed that the SOC enhancement program strengthened the self-efficacy, social support, and SOC in the elderly with metabolic syndrome, improved the health behavior, and was effective in improving some metabolic-related physiological indicators. Based on the results of this study, I suggest to expand the scope of the sense of coherence program and conduct the intervention longer.

